# Interactions between Angiotensin Type-1 Antagonists, Statins, and ROCK Inhibitors in a Rat Model of L-DOPA-Induced Dyskinesia

**DOI:** 10.3390/antiox12071454

**Published:** 2023-07-19

**Authors:** Andrea Lopez-Lopez, Rita Valenzuela, Ana Isabel Rodriguez-Perez, María J. Guerra, Jose Luis Labandeira-Garcia, Ana Muñoz

**Affiliations:** 1 Research Center for Molecular Medicine and Chronic Diseases (CIMUS), IDIS, University of Santiago de Compostela, 15782 Santiago de Compostela, Spain; andrealopez.lopez@usc.es (A.L.-L.); rita.valenzuela@usc.es (R.V.); anai.rodriguez@usc.es (A.I.R.-P.); mjosefanativid.guerra@usc.es (M.J.G.); 2 Networking Research Center on Neurodegenerative Diseases (CIBERNED), 28029 Madrid, Spain

**Keywords:** angiotensin, brain cholesterol, dyskinesia, fasudil, L-DOPA, neuroinflammation, oxidative stress, Parkinson, Rho-Kinase, simvastatin

## Abstract

Statins have been proposed for L-DOPA-induced dyskinesia (LID) treatment. Statin anti-dyskinetic effects were related to the inhibition of the Ras-ERK pathway. However, the mechanisms responsible for the anti-LID effect are unclear. Changes in cholesterol homeostasis and oxidative stress- and inflammation-related mechanisms such as angiotensin II and Rho-kinase (ROCK) inhibition may be involved. The nigra and striatum of dyskinetic rats showed increased levels of cholesterol, ROCK, and the inflammatory marker IL-1β, which were reduced by the angiotensin type-1 receptor (AT1) antagonist candesartan, simvastatin, and the ROCK inhibitor fasudil. As observed for LID, angiotensin II-induced, via AT1, increased levels of cholesterol and ROCK in the rat nigra and striatum. In cultured dopaminergic neurons, angiotensin II increased cholesterol biosynthesis and cholesterol efflux without changes in cholesterol uptake. In astrocytes, angiotensin induced an increase in cholesterol uptake, decrease in biosynthesis, and no change in cholesterol efflux, suggesting a neuronal accumulation of cholesterol that is reduced via transfer to astrocytes. Our data suggest mutual interactions between angiotensin/AT1, cholesterol, and ROCK pathways in LID, which are attenuated by the corresponding inhibitors. Interestingly, these three drugs have also been suggested as neuroprotective treatments against Parkinson’s disease. Therefore, they may reduce dyskinesia and the progression of the disease using common mechanisms.

## 1. Introduction

Parkinson’s disease (PD) is mainly characterized by the loss of dopaminergic neurons in the substantia nigra. The dopamine precursor, L-DOPA, is the most effective treatment for PD. Most patients develop L-DOPA-induced dyskinesia (LID), which consists of abnormal involuntary movements associated with rapid fluctuations in dopamine levels, limiting the effectivity of the treatment, particularly at advanced stages of the disease [1,2]. As multiple factors are involved, the pathophysiology of LID has not been totally clarified, which makes it difficult to achieve fully effective therapy [3]. Dysfunctional corticostriatal synapses [4], the abnormal release of dopamine by the serotonergic terminals [5,6], dysregulation in nitric oxide levels [7], neuroinflammation [8], and extracellular signal-regulated kinase (ERK) hyperactivation [9,10] have been related to LID. Different compounds acting on any of these factors have been suggested as potential therapies against LID. However, several of these factors may interact with each other.

Different studies have shown that neuroinflammation [8,11,12], inflammation-related angiogenesis, and the release of pro-inflammatory cytokines, particularly interleukin-1β (IL-1β), induce changes in blood–brain barrier (BBB) permeability and play a major role in LID development [13,14]. Consistent with this, the use of anti-inflammatory agents has been suggested as a potential therapy for LID [8]. In previous studies, we have shown that compounds blocking major mechanisms involved in the neuroinflammatory response led to a significant reduction in LID in the classic 6-hydroxydopamine (6-OHDA) rat model. First, we observed that antagonists of angiotensin type I (AT1) receptors such as candesartan reduce LID [15], which was recently supported by a retrospective case–control study [16]. AT1 is the main pro-oxidative/proinflammatory axis of the renin-angiotensin system (RAS). Angiotensin II (Ang II), via AT1 receptors, activates the NADPH-oxidase complex, which is the second most important source of superoxide in cells after mitochondria [17], leading to oxidative stress and inflammation if it is not properly counteracted by the RAS anti-oxidative axis [18,19]. The activation of the angiotensin II/AT1/NADPH-oxidase/superoxide axis induces Rho-kinase (ROCK) activation, which plays a major role in the RAS pro-inflammatory effects [20]. Both AT1 receptor activation [21,22,23] and ROCK activation [24,25] interact with each other to mediate the neuroinflammatory response, angiogenesis, and BBB permeability [20,26]. Consistent with this, we recently observed the potent antidyskinetic effect of ROCK inhibitors such as fasudil [21]. Fasudil reduced the development of L-DOPA-induced dyskinesia and inhibited already-established dyskinesia without affecting the therapeutic effect of L-DOPA (see [21] for details). In clinical practice, fasudil has been used against vascular diseases since 1995, and the safety and benefits of the compound are known [26,27].

Statins are inhibitors of the rate-limiting enzyme in cholesterol biosynthesis (3-hydroxy-3-methylglutarylcoenzyme A reductase; HMGCR). However, statins can also inhibit the Ras-ERK pathway, which has been related to dyskinesias [28], and have been suggested as a possible treatment against LID [28,29,30]. Statins have been observed to reduce both LID and ERK1/2 phosphorylation in rat and monkey models. However, simvastatin, which appears to be the most effective at crossing the BBB [31], was not effective in an exploratory trial in patients, which suggested that higher doses, unsafe in prolonged administration, were possibly necessary [28]. Additional trials have recently been considered (https://clinicaltrials.gov/ct2/show/NCT04064294, accessed on 5 May 2023). However, the effects of statins may be mediated by other mechanisms, such as AT1 and ROCK inhibition, and AT1 or ROCK inhibitors may substitute or enhance the effects of statins and reduce the toxic dose required for LID inhibition. Furthermore, it is not known whether statin-induced reduction in brain cholesterol levels and possible anti-inflammatory effects of statins are also involved in their anti-LID effects [32,33,34]. In the work reported here, we studied whether changes in ROCK and AT1 activity may mediate the effects of statins on LID, and the possible consequences on antidyskinetic therapeutical strategies.

## 2. Materials and Methods

### 2.1. Experimental Design

For in vivo experiments, we used adult female Sprague Dawley rats (weighing 225–250 g at the beginning of the experiments). Female rats were used because the important increase in body weight of male rats in long-term experiments may affect motor behavioral tests, and all rats selected for the experiments already had maximal dopaminergic denervation (i.e., levels of dopaminergic denervation are not influenced by estrogen levels). Furthermore, we used male mice deficient (KO) for AT1 or AT2 receptors with the corresponding wild-type (wt) controls: C57BL-6 wild-type (Charles River, L ‘Arbresle, France). Homozygous C57BL-6 mice deficient in AT1a (the major mouse AT1 isoform and the closest murine homolog to the single human AT1) were obtained from the Jackson Laboratory (Bar Harbor, ME, USA) and homozygous C57BL-6 mice deficient in AT2 receptors were a generous gift from Dr. Daniel Henrion. Our previous studies [35,36] on sex differences in AT1 and AT2 KO mice showed that changes in neuroinflammatory responses, such as those investigated in the present study using KO mice, are more easily detected in male mice, which constitute a more useful tool for this specific purpose and do not present the weight gain inconvenient of male dyskinetic rats. Rats and mice were housed under a 12 h light/dark cycle and with ad libitum access to food and water. Cell cultures from a neuron cell line (N27) and an astroglial cell (C6) were used for in vitro experiments. All experiments were carried out in accordance with the European Communities Council Directive 2010/63/EU, Directive 86/609/EEC, and Spanish RD 526/2014, and were approved by the corresponding committee at the University of Santiago de Compostela.

**Figure 1:**
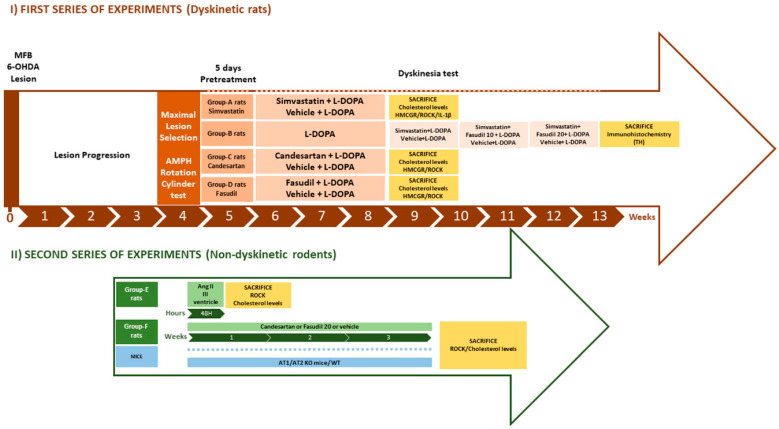
Experimental design of the in vivo experiments. Groups of rats and mice and time-course of experiments: lesions, behavioral tests, treatments, and sample analysis. Abbreviations: Amph, amphetamine; Ang II, angiotensin II; MFB, medial forebrain bundle; TH, tyrosine hydroxylase.

In an initial series of experiments (Figure 1), we investigated the effects of simvastatin in the development of LID in 6-OHDA-lesioned rats. Doses of L-DOPA and different antidyskinetic drugs were based on previous studies of our laboratory and others [15,21,30,37]. A first group of 6-OHDA-lesioned rats (group-A rats) were daily injected with L-DOPA (6 mg/kg/day; sc plus 10 mg/kg benserazide, a peripheral dopa decarboxylase inhibitor for 3 weeks) and we treated one subgroup of animals with simvastatin (15 mg/kg, orally) to test its effect on the development of LID (*n* = 10), and a subgroup was treated with vehicle (*n* = 10). Simvastatin or vehicle was given 5 days prior to the L-DOPA treatment and 1 h before each L-DOPA injection. These dyskinetic rats were also used to determine the effects of LID and simvastatin on nigral and striatal levels of cholesterol, the cholesterol biosynthesis marker HMGCR, ROCK activity, and markers of neuroinflammation such as IL-1β. A second group of rats (group-B rats) were used to investigate whether the same dose of simvastatin could reverse dyskinesia in L-DOPA primed rats (i.e., treated once the dyskinesia was consistently established). Animals were first injected with L-DOPA only (6 mg/kg/day, sc; plus 10 mg/kg of benserazide) for 3 weeks. Once LID was established, the rats were matched in 2 similar subgroups based on their dyskinesia scores, and the dyskinetic behavior was analyzed for periods of 10 days. One of the groups was treated with simvastatin (15 mg/kg, orally) and L-DOPA (*n* = 7), a control group was treated with vehicle + L-DOPA (*n* = 7), and the dyskinetic behavior was analyzed for an initial period of 10 days. In the second period, we co-treated the animals simultaneously with simvastatin and fasudil (10 mg/kg/day, ip, in saline; LC laboratories, Woburn, MA, USA) to test the synergistic effect between simvastatin and ROCK inhibition on LID. Finally, during a third period, this group of rats was co-treated with simvastatin and a higher dose of fasudil (20 mg/kg/day). A third group of dyskinetic rats (group C rats) were used to investigate the effect of AT1 receptor blockers on the LID-induced changes in the cholesterol biosynthesis marker HMGCR. Five days before L-DOPA treatment, rats were treated with the AT1 receptor antagonist candesartan (6 mg/kg/day orally; 1 h before each L-DOPA injection) (*n* = 6) or vehicle (*n* = 5).

Group D rats were used to investigate the effect of ROCK inhibitors on the LID-induced changes in the cholesterol biosynthesis marker HMGCR. Five days before L-DOPA treatment, rats were treated with the ROCK inhibitor fasudil (20 mg/kg/day, ip; 30 min before each L-DOPA injection; LC laboratories; *n* = 5) or vehicle (*n* = 5).

A second series of experiments (Figure 1) was designed to further investigate the interaction between cholesterol, ROCK, and angiotensin II (Ang II) observed in the above-mentioned experiments. In a group of rats (group E rats), we studied the effect of the Ang II injected into the third ventricle on the nigral and striatal levels of cholesterol and ROCK (*n* = 7) in comparison with control rats injected with vehicle (*n* = 7). An additional group of rats (group F rats) was treated with the Ang II blocker candesartan (6 mg/kg, orally; *n* = 6) or the ROCK inhibitor fasudil (20 mg/kg ip; *n* = 6) or vehicle (*n* = 5) over 3 weeks to observe effects on levels of ROCK and cholesterol. Furthermore, we analyzed changes in ROCK and cholesterol levels in the substantia nigra and striatum of mice deficient for AT1 (*n* = 6) or AT2 (*n* = 6) receptors compared to the corresponding wt controls (*n* = 12).

At the end of the experiments, the rats (Groups A, C–F) and mice were stunned with carbon dioxide and then killed by decapitation (90 min after the last L-DOPA injection or 48 h after the injection of Ang II in the third ventricle). The substantia nigra and the striatum were rapidly dissected on ice, and stored at −80 °C until processed. Rats in group B were processed for tyrosine hydroxylase immunohistochemistry.

Finally, we performed in vitro experiments using a dopaminergic neuron cell line (N27) and an astroglial cell line (C6) to identify cells and mechanisms involved in changes in cholesterol levels observed in the above-mentioned in vivo experiments.

### 2.2. 6-Hydroxidopamine Lesion of the Dopaminergic System

Surgery was performed on rats deeply anesthetized with a mixture of ketamine (50 mg/kg)/medetomidine (0.4 mg/kg) and mounted in a stereotaxic frame (Kopf Instruments, Tujunga, CA, USA). Subcutaneous buprenorphine (0.05 mg/kg) was administrated for analgesia at the end of the surgery, immediately after the incision was closed, and before the rat regained consciousness. A second dose was administered 12 h later. Lesions were produced in the right medial forebrain bundle to achieve the complete lesions of the nigrostriatal pathway. The rats were injected with 12 µg of 6-hydroxidopamine (6-OHDA; to provide 8 µg of 6-OHDA free base; Sigma, St. Louis, MO, USA) in 4 µL of sterile saline containing 0.2% ascorbic acid. The stereotaxic coordinates were 3.7 mm posterior to bregma, 1.6 mm lateral to the midline, and 8.8 mm ventral to the skull at the midline in the flat skull position. The tooth bar was set at −3.3 mm. The solution was injected using a 5 µL Hamilton syringe coupled to a motorized injector (Stoelting), at a rate of 0.5 µL/min, and the needle was left in situ 2 min after injection. Four weeks post-surgery, the efficacy of the lesion was evaluated via the amphetamine rotation test and the cylinder test (see below). Rats showing maximal dopaminergic denervation (>90%), identified with the behavioral tests, were included in the study. The extent of the lesion was finally verified using tyrosine hydroxylase Western blot or immunohistochemistry analysis. Female rats were used for chronic experiments as in previous studies for dyskinesia experiments, as explained above [15,21].

### 2.3. Intraventricular Angiotensin II Injections

A group of adult Sprague Dawley rats was injected in the third ventricle with a single dose of 5 μg of Ang II in 3 μL sterile saline or sterile saline alone as a control group to study the effects of Ang II on brain cholesterol levels. The Ang II-injected dose was determined based on our previous studies [15,38].

The surgical procedure was similar to that performed in 6-OHDA lesions. The stereotaxic coordinates were −0.8 mm posterior to bregma and −6.5 mm ventral to the skull at the midline in the flat skull position. The tooth bar was set at 0 mm. The solution was injected using a 5 µL Hamilton syringe coupled to a motorized injector (Stoelting, Wood Dale, IL, USA), at a rate of 0.5 µL/min, and the needle was left in situ 2 min after injection. After 48 h, rats were stunned with carbon dioxide and killed via decapitation. Ventral mesencephalon and striatum brain areas were dissected for the measurements of cholesterol levels.

### 2.4. Behavioral Analysis

#### 2.4.1. Cylinder Test

The cylinder test was used to evaluate forelimb akinesia and the efficacy of the lesion [39]. Rats were placed into a transparent cylinder, 20 cm in diameter, and videoed. The cylinder had two mirrors, enabling animal display from all directions, including when they turned back. The rats could move freely within the cylinder, exploring the environment. Until a total of 20 touches, the number of weight-bearing touches of each forelimb was counted by a researcher blinded to the treatment. The results were shown as the percentage of touches made with the lesioned paw relative to the total number of touches. A control rat should present a score of 50% of the total counts (dashed line in the figures), while the lesion should reduce the touches of the injured paw below 20%. The cylinder test was also used to study the possible effects of simvastatin on motor improvement induced via L-DOPA treatment. The baseline value was calculated for each session before any injection (i.e., off-drug), and 90 min after the L-DOPA injection, the rats were tested again. Simvastatin was administrated 1 h before L-DOPA injection.

#### 2.4.2. Amphetamine- and L-DOPA-Induced Rotation

Rotational behavior induced by amphetamine was tested 4 weeks after the 6-OHDA injection to assess the extent of the dopaminergic lesion. Rotations were measured using an automated rotometer (Rota-count 8, Columbus Instruments, Columbus, OH, USA). Left and right full body turns induced by 2.5 mg/kg (in saline) amphetamine intraperitoneal injection were counted over a 90 min period. Rats showing rotations higher than 6 net full turns per minute towards the lesioned side were identified as maximally lesioned rats (i.e., showing more than 90% depletion of striatal dopaminergic terminals) [40] and were included in the study.

#### 2.4.3. L-DOPA-Induced Dyskinesia

In order to produce L-DOPA-induced dyskinesia (LID), L-DOPA (6 mg/kg) was administered daily to each rat as a subcutaneous injection for 3 weeks. L-DOPA was administered together with benserazide (peripheral DOPA decarboxylase inhibitor; 10 mg/kg, in saline). The evaluation of LID was performed following the rat dyskinesia scale [15,21]. Briefly, the animals were placed in individual transparent plastic cages without bedding material and were scored every 20 min after the injection of L-DOPA and for the entire time course of dyskinesias. The abnormal involuntary movements (AIMs) were classified into four subtypes, according to their topographic distribution as limb (purposeless movements of the contralateral forelimb), orolingual (empty jaw movements and contralateral tongue protrusion), axial (dystonic posturing or twisting movements of the neck and the upper part of the body towards the side contralateral to the lesion), and locomotive movement (circling movements away from the lesioned side). The forelimb and orolingual dyskinesia are predominantly seen as hyperkinesia, while axial dyskinesia is essentially a dystonic movement. The rating did not include enhanced manifestations of normal behaviors, such as grooming, gnawing, rearing, and sniffing. The severity of each AIM subtype was assessed using scores from 0 to 4 (1: occasional, i.e., present <50% of the time; 2: frequent, i.e., present >50% of the time; 3: continuous but interrupted by strong sensory stimuli; 4: continuous, not interrupted by strong sensory stimuli). Integrated AIM scores and each AIM subtypes were shown as time-course curves. Raw data plot of total scores: limb + orolingual + axial AIM scores multiplied by the observation interval (i.e., 20 min).

### 2.5. Western Blot

Tissue from rat ventral midbrain or striatum and C6 or N27 cells were lysed in RIPA buffer containing protease inhibitor cocktail (Sigma-Aldrich) and PMSF (Sigma-Aldrich). Lysates were centrifuged and proteins were quantified using the Pierce BCA Protein Assay kit (Thermo Fisher Scientific, Inc., Waltham, MA, USA). An equal amount of protein lysates was separated on an 8% Bis-Tris polyacrylamide gel and transferred to nitrocellulose membranes. Membranes were incubated overnight with primary antibodies against proteins of interest: rabbit polyclonal anti-ABCA1 transporter (ab7360; 1:1000, Abcam, Cambridge, UK), rabbit polyclonal anti-LDL receptor, LDLR (ab30532; 1:1000, Abcam), rabbit monoclonal anti-HMGCR enzyme (ET1702-41; 1:1000, Huabio, Woburn, MA, USA), Armenian hamster monoclonal anti-IL-1β (sc-12742; 1:100, Sta. Cruz Biotechnology, Santa Cruz, CA, USA), or mouse monoclonal anti-ROCK-2 protein (sc-398519; 1:200, Sta. Cruz Biotechnology). Mouse anti-tyrosine hydroxylase antibody (TH, Sigma-Aldrich Cat# T2928, RRID: AB_477569; 1:5000) was also used to confirm the dopamine denervation.

Blots were reproved with HRP anti-alpha tubulin (ab185067; 1:5000, Abcam) or GAPDH (G9545; 1:25,000, Sigma-Aldrich) as a loading control. The membranes were incubated with the following HRP-conjugated secondary antibodies: goat anti-rabbit (1:2500) and HRP-conjugated rabbit anti-mouse (1:2500) from Agilent. Immunoreactive bands were detected with an Immun-Star HRP Chemiluminescent Kit (170-5044; Bio-Rad, Hercules, CA, USA) and visualized with a chemiluminescence detection system (Molecular Imager ChemiDoc XRS System; Bio-Rad). The data were then expressed relative to the value obtained for the control (100%) to counteract possible variability among batches. Finally, the results were expressed as means ± SEM.

### 2.6. Real-Time PCR Analysis

Nigral and striatal total ribonucleic acid (RNA) was extracted using the Trizol method (following the manufacturer’s instructions). We used a NanoQuant plate and an Infinite M200 multiwell plate reader (TECAN, Salzburg, Austria) to determine the RNA concentration. We reverse-transcribed the total RNA (2 µg) to complementary DNA (cDNA) using random primers, deoxynucleotide triphosphate (dNTP), and Moloney murine leukemia virus reverse transcriptase (M-MLV; 200 U, Invitrogen). The relative levels of ABCA1, LDLR mRNA, and HMGCR messenger RNA (mRNA) were determined using real-time PCR. β-actin was used as a housekeeping gene and was amplified in parallel with the genes of interest. Forward (F) and reverse (R) primers were designed for each gene by using Beacon Designer software (Premier Biosoft, Palo Alto, CA, USA). Forward (F) and reverse (R) primers were designed for each gene by using NCBI Primer-BLAST (https://www.ncbi.nlm.nih.gov/tools/primer-blast accessed on 4 May 2022), primers were located on the exon–exon junction to avoid the amplification of genomic DNA. Primer sequences were as follows: for HMGCR, forward 5′-AACTCACAGGATGAAGTAAG-3′, reverse 5′-AAAGCAGCACATGATCTCAA-3′; for ABCA1, forward 5′-GGAGCTCTTTACAAACAATAAAT-3′, reverse 5′-CAAAAAGGTTCCGTCCTACT-3′; for LDLR, forward 5′GAACTCTGTTCCGAGAGAAA-3′, reverse 5′-CCACTGGGAAGATCTAGTGT-3′; and for β-actin, forward 5′-TCGTGCGTGACATTAAAGAG-3′, reverse 5′-TGCCACAGGATTCCATACC-3′. Experiments were performed using a QuantStudio 3 platform (Applied Biosystems, Foster City, CA, USA), the EvaGreen qPCR MasterMix (Applied Biological Materials Inc., Vancouver, Canada). The data were evaluated via the delta–delta Ct method (2^−ΔΔCt^), where Ct is the cycle threshold. The expression of each gene was obtained relative to the housekeeping transcripts. The data were then normalized to the values of the control group and the results were expressed as mean ± standard error of the mean (SEM).

### 2.7. Amplex Red Cholesterol Assay

Cholesterol levels were measured using the commercial kit Amplex Red Cholesterol Assay (A12216, Molecular Probes, Invitrogen, Thermo Fisher Scientific, Waltham, MA, USA), according to the manufacturer’s instructions. The tissue samples were homogenized in RIPA buffer containing protease inhibitor cocktail (Sigma) and PMSF (Sigma). Next, samples were centrifuged 14,000× *g* for 20 min and the resultant pellet was resuspended in quantification buffer. A total of 50 μL of cholesterol standards (0–8 μg/mL) or sample duplicates (10 µg) were added to 96-well black plates. Then, 50 μL of a working solution containing 300 μM Amplex Red probe with 2 U/mL of horseradish peroxidase, 2 U/mL cholesterol oxidase, and 0.2 U/mL of cholesterol esterase was added. The plate was incubated for 30 min at 37 °C, protected from light, and monitored at excitation/emission = 560/590 nm using an Infinite M200 multiwell plate reader (TECAN). Final cholesterol content was expressed as μg/mL cholesterol/μg of protein tissue.

### 2.8. ROCK Activity Assay

ROCK activity was measured with a ROCK Activity Assay kit (CellBiolabs, Inc, San Diego, CA, USA) according to the manufacturer’s instructions. The ROCK Activity Assay kit is an enzyme immunoassay developed for the detection of the specific phosphorylation of myosin phosphatase target subunit 1 at Thr696 by ROCK. Tissue was homogenized in lysis buffer (50 mM Tris–HCl pH 7.5, 150 mM NaCl, 1 mM beta-glycerolphosphate, 1% Triton X-100, 1 mM EDTA, 1 mM EGTA) 1 mM NA_3_VO_4_ containing protease inhibitor cocktail (P8340, Sigma). The protein concentration of extracts was measured with the Pierce BCA Protein Assay Kit (Thermo Fisher Scientific, Inc., Waltham, MA, USA) and equal amounts of protein (5 μg per well) were used; each sample was assayed in duplicate. Phosphorylation activity was assessed by measuring the absorbance at 450 nm in an Infinite M200 multiwell plate reader (TECAN).

### 2.9. Immunohistochemistry

Rats processed for immunohistochemistry were perfused with 0.9% saline and then with cold 4% paraformaldehyde in 0.1 M phosphate buffer, pH 7.4. The brain was removed and then washed and cryoprotected in the same buffer containing 20% sucrose. Brains were then cut into 40 μm sections on a freezing microtome. The sections were incubated for 1 h in 10% normal swine serum with 0.25% Triton X-100 in 20 mM potassium phosphate-buffered saline, containing 1% bovine serum albumin (KPBS-BSA), and subsequently incubated overnight (at 4 °C) with anti-tyrosine hydroxylase (TH) as the dopaminergic marker (mouse monoclonal anti-TH, Sigma-Aldrich Cat# T2928, RRID: AB_477569; 1:10,000). Sections were then incubated with the corresponding biotinylated secondary antibody (horse anti-mouse, Vector Laboratories, Inc., Newark, CA, USA; Cat# BA-2001, RRID: AB_2336180; 1:200) for 60 min, and then with an avidin–biotin–peroxidase complex (ABC, 1:100, Vector) for 90 min. Finally, sections were revealed with 0.04% hydrogen peroxide and 0.05% 3-3′diaminobenzidine (DAB, D5637, Sigma-Aldrich). All experiment control sections in which the primary antibody was omitted were immune-negative for TH.

### 2.10. Cell Line Cultures

The C6 astroglial cells (Sigma-Aldrich) were cultured in Ham’s F12 medium with 10% FBS, 2 mM L-glutamine (Sigma-Aldrich), 100 U/mL penicillin, and 100 μg/mL streptomycin. The N27 dopaminergic neuron cell line was cultured in RPMI 1640 medium supplemented with 10% FBS, 2 mM L-glutamine (Sigma-Aldrich), 100 U/mL penicillin, and 100 μg/mL streptomycin. Cultures were maintained at 37 °C and 5% CO_2_ in a humidified incubator. Cells were plated at a density of 5 × 10^5^ cells/cm^2^ onto 35 mm culture dishes plates. At 24 h after seeding, cells were treated with Ang II for another 24 h, and protein and total RNA were extracted for protein analysis via WB and mRNA expression using RT-PCR. The effective doses of Ang II were determined based on our previous findings; C6 astroglial cells were treated with 100 nM Ang II, while dopaminergic N27 cells with a concentration of 1 µM Ang II due to the differences in angiotensin receptors expression between both cell cultures [41].

### 2.11. Statistical Analysis

All data were obtained from at least three independent experiments and expressed as means ± SEM. Student’s *t*-test was used for two-group comparisons. One-way ANOVA followed by a post hoc Holm–Sidak test was used to analyze multiple comparisons. Two-way repeated measure (RM) ANOVA (time and treatment as factors) followed by Holm–Sidak post hoc comparisons was used to analyze dyskinesia data. Relevant pairwise differences were confirmed by the non-parametric Mann–Whitney U-test. The normality of populations and the homogeneity of variances were analyzed before each test. Differences were considered statistically significant at *p* < 0.05. All statistical analyses were carried out using SigmaPlot 11.0 (Jandel Scientific, Systat Software, Inc., San Jose, CA, USA).

## 3. Results

### 3.1. Simvastatin Reduces the Development of L-DOPA-Induced Dyskinesia

**Figure 2:**
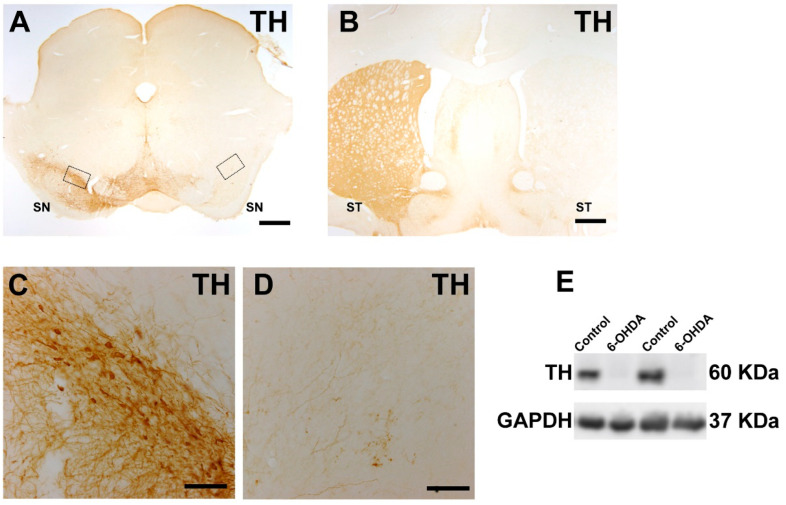
A maximal lesion (i.e., lack of TH-immunoreactivity) can be observed by immunohistochemistry in the right substantia nigra (**A**) and right striatum (**B**) relative to the non-lesioned side (left side) or through Western blot analysis comparing the control side and the lesioned side (6-OHDA); (**E**) Nigral areas squared in A were magnified in (**C**,**D**). Abbreviations: SN, substantia nigra compacta; ST, striatum; TH, tyrosine hydroxylase. Scale bars: 600 µm (**A**,**B**); 100 µm (**C**,**D**).

As shown in many previous studies, rats with maximal dopaminergic lesions induced through unilateral 6-OHDA injection and selected using the above-described behavioral criteria showed an almost total loss of dopaminergic neurons in the ipsilateral substantia nigra and dopaminergic terminals in the ipsilateral striatum, as observed via TH-immunohistochemistry, as well as an almost total loss of TH protein, as observed via Western blot (Figure 2).

**Figure 3:**
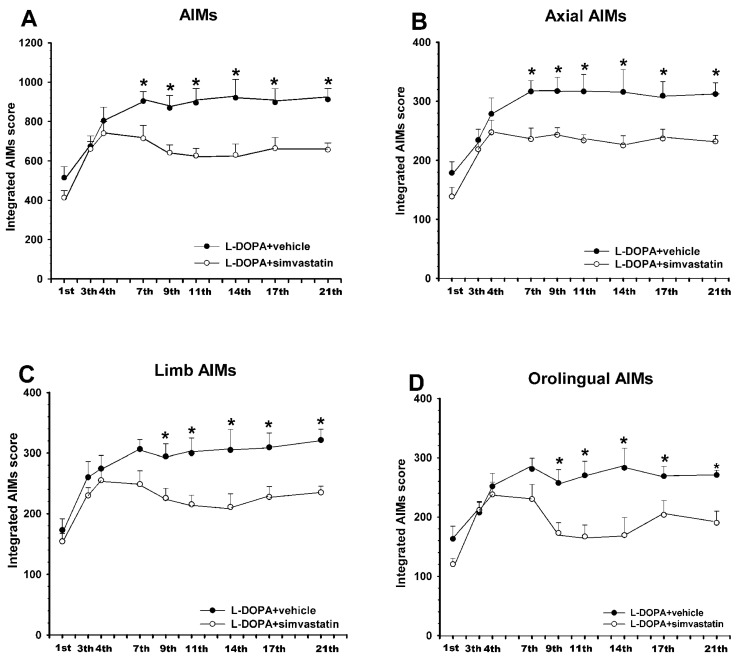
Effect of simvastatin on the development of L-DOPA-induced dyskinesia. Rats co-treated with the HMGCR inhibitor, simvastatin (15 mg/kg, orally), and L-DOPA (6 mg/kg; white circles) for 3 weeks showed a significant reduction in the development of AIMs relative to control rats treated with vehicle and L-DOPA (dark circles). This decrease was observed in the AIMs total score (**A**) and in the different components: axial (**B**), limb (**C**), and orolingual (**D**). Total AIMs score (**A**) was estimated as the addition of limb (**C**), orolingual (**D**), and axial (**B**) components. Values are expressed as means ± SEM. Two-way ANOVA for repeated measures and Holm–Sidak post hoc test, *, *p* < 0.05. AIMs, abnormal involuntary movements.

**Figure 4:**
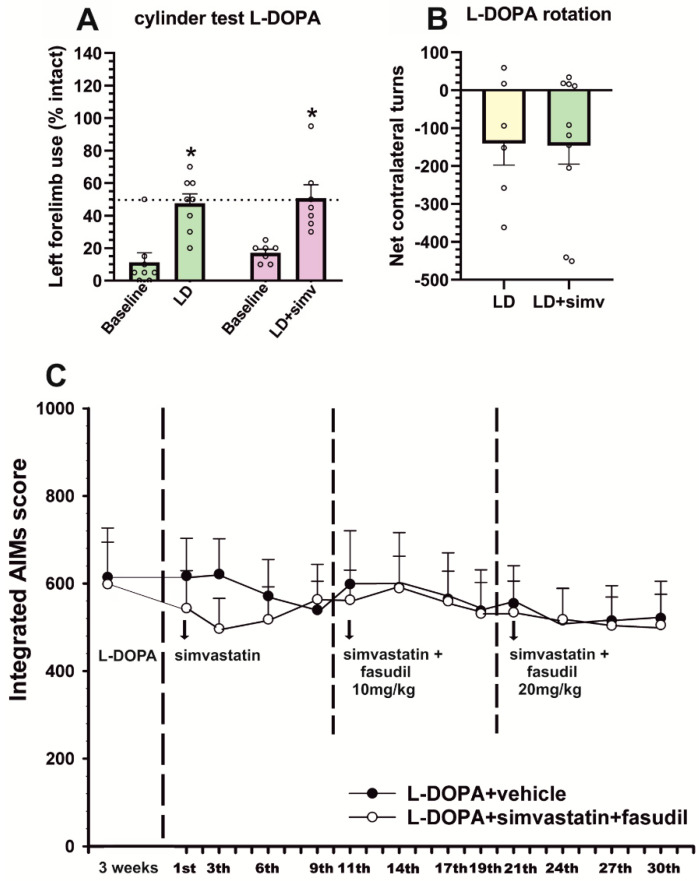
Effect of simvastatin on the therapeutic effect of L-DOPA assessed via the cylinder test and turning behavior (**A**,**B**) and the effect of simvastatin and the co-treatment of simvastatin and the ROCK inhibitor fasudil in L-DOPA-primed rats (**C**). (**A**) Spontaneous forelimb use in the cylinder test revealed that simvastatin administration did not reduce the ability of L-DOPA to improve forelimb akinesia, showing a similar number of touches performed with the left paw in animals treated with simvastatin and L-DOPA (LD + simv; violet bars) than in those treated with vehicle and L-DOPA (LD; green bars). (**B**) Total contralateral turns in 90 min showed that simvastatin did not reduce contralateral rotation. (**C**) In rats with previously established L-DOPA-induced dyskinesia (L-DOPA-primed rats, treated with L-DOPA 6 mg/kg for 3 weeks), treatment with oral simvastatin (15 mg/kg; white circles) and L-DOPA did not induce any significant difference in AIMs score with respect to control rats treated with vehicle and L-DOPA (dark circles). Co-treatment with simvastatin and fasudil (10 or 20 mg/kg) did not induce any significant improvement in dyskinetic behavior. In A and B, data are means ± SEM. * *p* < 0.05, significant differences relative to baseline levels, off L-DOPA, Student *t*-test. In (**C**), two-way ANOVA for repeated measures and Holm–Sidak post hoc test, * *p* < 0.05. AIMs, abnormal involuntary movements.

6-OHDA-lesioned rats injected daily with L-DOPA for 3 weeks showed an intense dyskinetic behavior. Treatment with simvastatin induced a significant reduction in the dyskinesia scores relative to rats treated with L-DOPA only. These differences between groups were consistently observed from the 7th L-DOPA injection until the end of the treatment period (21 days, 3 weeks). The analysis of different components showed a significant reduction in the axial (around 25% reduction), limb (around 25% reduction), and orolingual (around 30% reduction) components (Figure 3). To investigate the impact of the simvastatin treatment on the therapeutic effect of L-DOPA, we performed a cylinder test after the second L-DOPA injection. Results revealed no differences in the improvement of left paw use relative to baseline scores in the animals treated with simvastatin relative to animals treated with L-DOPA only, demonstrating that simvastatin did not affect the motor recovery induced by L-DOPA (Figure 4A). Moreover, L-DOPA-induced rotation showed no differences in the group treated with simvastatin relative to those treated with L-DOPA only (showing an average of around 140 contralateral turns in 90 min in both groups, Figure 4B). This indicates that the observed decrease in LID was not a consequence of a simvastatin-induced reduction in motor activation.

### 3.2. Simultaneous Treatment with Low Doses of Fasudil Did Not Improve the Therapeutic Effects of Simvastatin on Already-Established Dyskinesia

When rats with already established dyskinesia were treated with the same dose of simvastatin (15 mg/kg, orally) for a period of 10 days, the decrease in dyskinetic behavior was not significant (Figure 4C). To uncover whether simultaneous treatment with the ROCK inhibitor fasudil could act synergistically with simvastatin to reduce dyskinesia, we added a low dose of fasudil (10 mg/kg) for a similar period. However, we did not observe any significant improvement. Then, the dose of fasudil was increased to 20 mg/kg for another period of 10 days, but no significant improvement was observed (Figure 4C). We did not further increase the dose of simultaneous treatment with fasudil because our previous studies showed that higher doses of fasudil alone can reduce already-established dyskinesia by themselves [21].

### 3.3. Dyskinetic Rats Showed Increased Levels of Cholesterol and Cholesterol Biosynthesis Marker HMGCR, ROCK Activity, and Interleukin-1β, Which Were Reduced by Simvastatin

**Figure 5:**
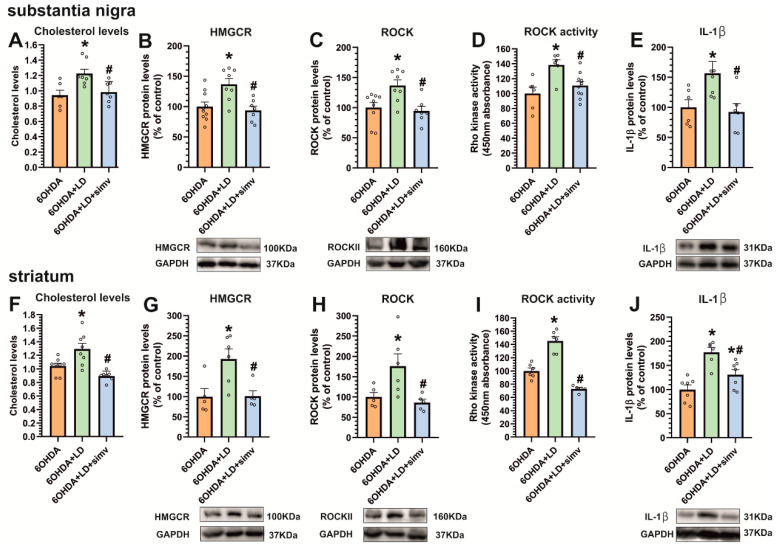
Effect of L-DOPA-induced dyskinesia and simvastatin on HMGCR, ROCK, and interleukin-1β. Dyskinetic rats chronically treated with L-DOPA 6 mg/kg (6-OHDA + LD, green bars) showed a significant increase in cholesterol levels (**A**,**F**) and in HMGCR protein levels (**B**,**G**), relative to 6-OHDA-lesioned rats (6-OHDA, orange bars) in the substantia nigra (**A**,**B**) and striatum (**F**,**G**). Moreover, dyskinetic animals showed a significant increase in ROCK protein levels (**C**,**H**), ROCK activity (**D**,**I**), and interleukin-1β (IL-1β) protein expression (**E**,**J**) in both regions. The inhibition of cholesterol biosynthesis by simvastatin (6-OHDA + LD + simv, blue bars) reduces dyskinesia, ROCK levels (**C**,**H**), ROCK activity (**D**,**I**), and IL-1β levels (**E**,**J**) in the striatum and substantia nigra. Data are means ± SEM. The results were normalized to the values of 6-OHDA-lesioned rats treated with vehicle. * *p* < 0.05, significant differences relative to 6-OHDA-lesioned rats; # *p* < 0.05 significant differences relative to L-DOPA-treated rats. One-way ANOVA and Holm–Sidak post hoc tests were used.

Dyskinetic animals (i.e., 6-OHDA-lesioned rats treated with L-DOPA) showed an increase in the cholesterol levels and expression of HMGCR in the substantia nigra and striatum, in comparison with 6-OHDA-lesioned rats treated with vehicle (Figure 5A,B,F,G). L-DOPA treatment also induced a significant increase in ROCK expression, ROCK activity, and interleukin-1β protein expression in the substantia nigra (Figure 5C–E) and striatum (Figure 5H–J). The inhibition of the HMGCR enzyme in animals treated with simvastatin induced a significant decrease in cholesterol and HMGCR levels in the nigra and striatum (Figure 5A,B,F,G). Interestingly, simvastatin also induced a significant decrease in ROCK protein expression and ROCK activity in the substantia nigra (Figure 5C,D) and striatum (Figure 5H,I). Moreover, the interleukin-1β analysis revealed a significant decrease in the nigra and striatum after simvastatin treatment (Figure 5E,J), suggesting that a reduction in LID-induced cholesterol levels, ROCK inhibition, and reduction in neuroinflammation are involved in the simvastatin-induced inhibition of dyskinesia.

### 3.4. Dyskinesia-Induced Increase in the Cholesterol Biosynthesis Marker HMGCR Is Reduced by ROCK Inhibitors and AT1 Receptor Blockers

**Figure 6:**
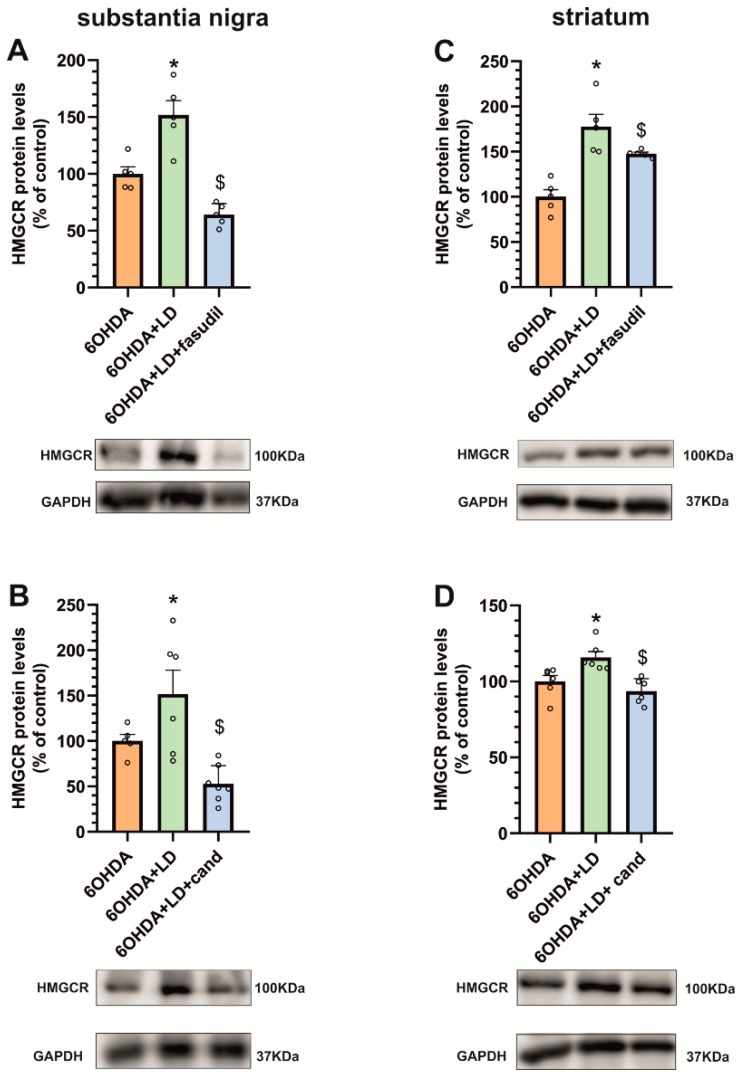
Effects of ROCK inhibitors and AT1 receptor blockers in the cholesterol biosynthesis marker HMGCR. The increase in HMGCR protein levels induced by dyskinesia (6-OHDA + LD, green bars) is reduced via the co-administration of L-DOPA and the ROCK inhibitor fasudil, (6-OHDA + LD + fasudil, blue bars) in the substantia nigra (**A**) and the striatum (**C**). Co-treatment with L-DOPA and the AT1 receptor antagonist candesartan (6-OHDA + LD + candesartan, blue bars) also produces a significant reduction in HMGCR protein levels induced through dyskinesia in the substantia nigra (**B**) and the striatum (**D**). The results were normalized to the values of 6-OHDA-lesioned animals treated with saline. Data are mean ± standard error of the mean (SEM); * *p* < 0.05, significant differences relative to 6-OHDA-lesioned rats; $ *p* < 0.05 significant differences relative to L-DOPA-treated rats. One-way ANOVA and Holm–Sidak post hoc test were used.

In dyskinetic rats treated with the ROCK inhibitor fasudil, we observed that the reduction in the dyskinetic behavior (see [21] for details) was accompanied by a significant reduction in HMGCR levels both in the substantia nigra and striatum (Figure 6A,C). Similarly, dyskinetic rats treated with the AT1 receptor antagonist candesartan showed a reduction in the dyskinetic behavior (see [15] for details) together with a significant reduction in HMGCR levels in the substantia nigra and striatum (Figure 6B,D).

### 3.5. AT1 Receptor Activity Induces the Increase in Cholesterol and ROCK Levels in the Striatum and Substantia Nigra

We studied whether the above-observed fasudil- and candesartan-induced decrease in cholesterol/HMGCR levels (Figure 6) may be an indirect effect of the drug-induced decrease in the dyskinetic response, as LID increased cholesterol levels. Therefore, we treated non-dyskinetic rats with Ang II, AT1 blockers, or the ROCK inhibitor fasudil to observe the direct effects of the inhibitors in the absence of LID.

**Figure 7:**
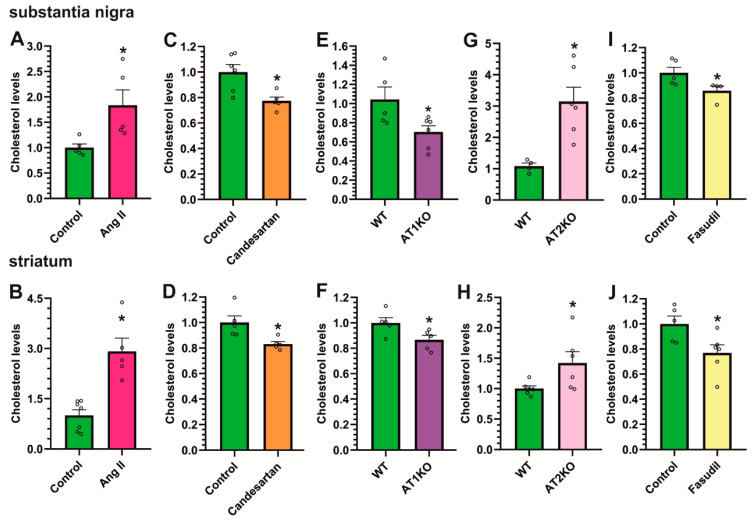
Effects of angiotensin, AT1 inhibition or AT1 or AT2 receptor deletion, and ROCK inhibition on cholesterol levels. Rats treated with an intraventricular injection of Angiotensin II (Ang II, pink bars) showed significantly higher levels of cholesterol in both substantia nigra (**A**) and striatum (**B**) compared to control saline-injected rats (green bars). The administration of the AT1 receptor antagonist candesartan (orange bars) induced a significant reduction in cholesterol levels in both regions (**C**,**D**). Moreover, AT1 KO mice (AT1KO, purple bars) showed significantly lower cholesterol levels than WT mice in the substantia nigra (**E**) and striatum (**F**), while AT2 KO mice (AT2KO, violet bars) showed significantly higher cholesterol content than in WT mice (**G**,**H**) in both regions. ROCK inhibition by fasudil (yellow bars) also induced a significant reduction in cholesterol levels in the nigra (**I**) and striatum (**J**). The results were normalized to the values of control animals treated with saline or WT mice. Data are mean ± standard error of the mean (SEM). * *p* < 0.05 compared to control (Student’s *t*-test).

First, we injected 5 μg Ang II into the third ventricle of normal rats. After 48 h, Ang II-injected rats showed significantly higher levels of cholesterol both in the substantia nigra and striatum (Figure 7A,B) relative to saline-injected controls. Consistent with this, the treatment of rats with the AT1 receptor antagonist candesartan induced a significant decrease in cholesterol levels in the nigra and striatum (Figure 7C,D), revealing the effect of AT1 receptor activity on cholesterol levels. In addition, AT1 or AT2 KO mice were used to further confirm the effects of Ang II major receptors on cholesterol content in the nigra and striatum. Consistent with that observed using candesartan, the nigra and striatum from AT1 KO mice showed significantly lower cholesterol levels than those of WT mice (Figure 7E,F). Furthermore, the nigra and striatum from AT2 KO mice, which are known to have AT1 receptor overactivity [35], showed higher cholesterol content than WT mice (Figure 7G,H), confirming the link between brain RAS and brain cholesterol content in vivo and the enhancing effects of AT1 receptor activity on cholesterol levels.

**Figure 8:**
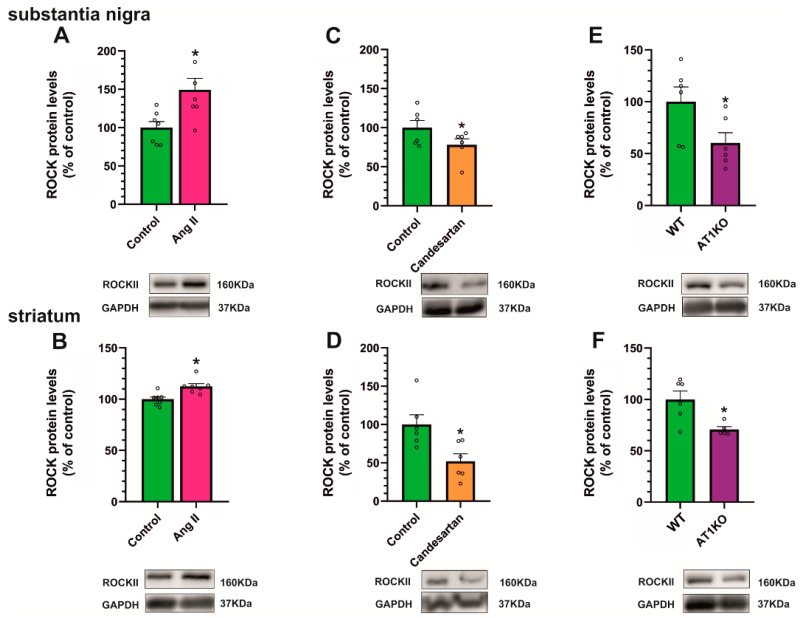
Effects of angiotensin, AT1 inhibition, or AT1 or AT2 receptor deletion on ROCK expression. Rats treated with intraventricular angiotensin II (Ang II, pink bars) showed a significant increase in ROCK protein expression levels in the substantia nigra (**A**) and striatum (**B**) relative to control rats injected with saline (green bars). However, treatment with the AT1 antagonist candesartan (orange bars) showed a significant reduction in ROCK levels in the substantia nigra (**C**) and the striatum (**D**). Consistent with this, AT1 KO mice (AT1KO, purple bars) showed a significant decrease in ROCK levels in comparison with control WT mice in both regions (**E**,**F**). The results were normalized to the values of control animals treated with saline or WT mice. Data are mean ± standard error of the mean (SEM). * *p* < 0.05 compared to control (Student’s *t*-test).

The treatment of rats with the ROCK inhibitor fasudil also induced a significant decrease in cholesterol levels in the nigra and striatum (Figure 7I,J), revealing interactions between cholesterol, Ang II/AT1, and ROCK pathways. To further confirm Ang II/ROCK interactions, we analyzed ROCK levels in the nigra and striatum of the above-mentioned rats and mice. Intraventricular treatment with Ang II also induced a significant increase in ROCK levels in the nigra and striatum (Figure 8A,B). Furthermore, ROCK levels were reduced in rats treated with the AT1 receptor blocker candesartan (Figure 8C,D) and AT1 KO mice (Figure 8E,F), suggesting that the effect is mediated by AT1 receptors.

### 3.6. Angiotensin II Increases Cholesterol Biosynthesis and Efflux in Dopaminergic Neuronal Cultures, While Increasing Cholesterol Uptake in Astrocyte Cultures

To study possible mechanisms involved in the above-mentioned interactions, we used in vitro models. Neurons and astrocytes are particularly involved both in brain cholesterol homeostasis and neuroinflammatory and neurodegenerative responses. Therefore, we used the cultures of the N27 dopaminergic neuron cell line and the C6 astrocyte cell line to study possible mechanisms responsible for the effects of Ang II on cholesterol levels.

**Figure 9:**
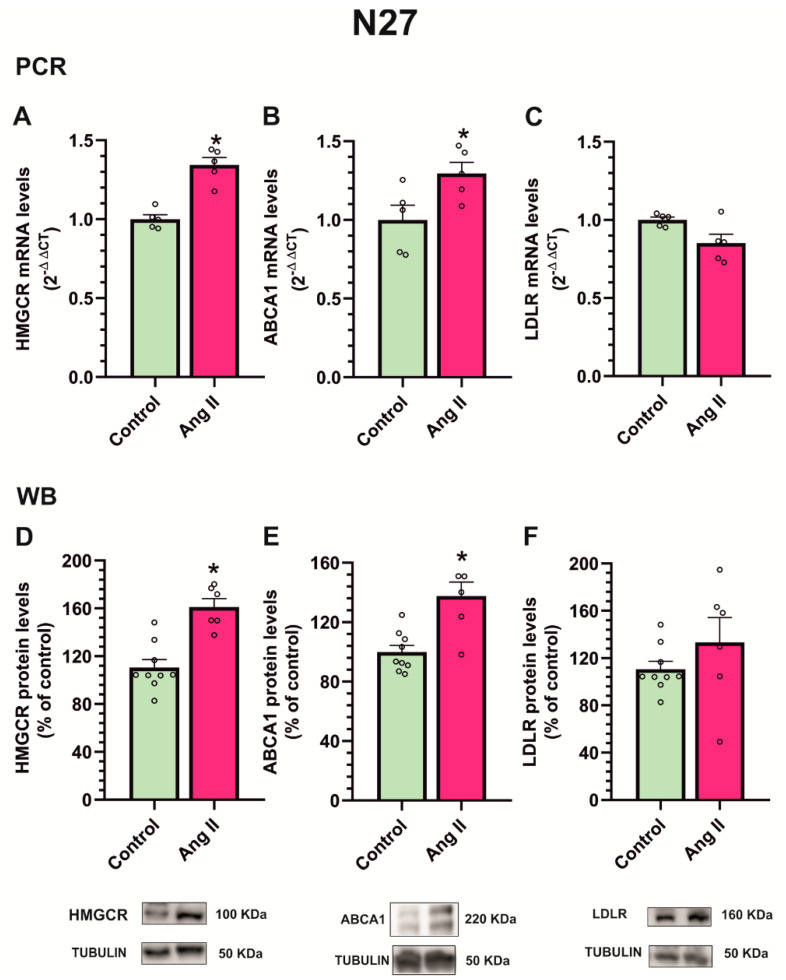
Effect of angiotensin treatment on N27 dopaminergic neuron culture. The treatment of N27 dopaminergic cell line with angiotensin II (Ang II) for 24 h produced an increase in the mRNA (**A**) and protein expression (**D**) of cholesterol biosynthesis enzyme HMGCR, an increase in cholesterol in efflux-related molecule ABCA1 (**B**,**E**), and no changes in cholesterol uptake-related molecule LDL receptor (LDLR) expression (**C**,**F**). Data are mean ± standard error of the mean (SEM). * *p* < 0.05 compared to control (Student’s *t*-test). HMGCR, 3-hydroxy-3-methylglutaryl CoA reductase; ABCA1, ATP-binding cassette transporter-1.

The treatment of the N27 dopaminergic cell line with Ang II for 24 h produced an increase in gene expression and protein levels of the cholesterol biosynthesis enzyme HMGCR (Figure 9A,D). Furthermore, we observed an increase in cholesterol efflux-related molecule ATP-binding cassette transporter-1 (ABCA1) (Figure 9B,E), while cholesterol uptake-related molecule LDL receptor (LDLR) expression did not significantly change (Figure 9C,F).

**Figure 10:**
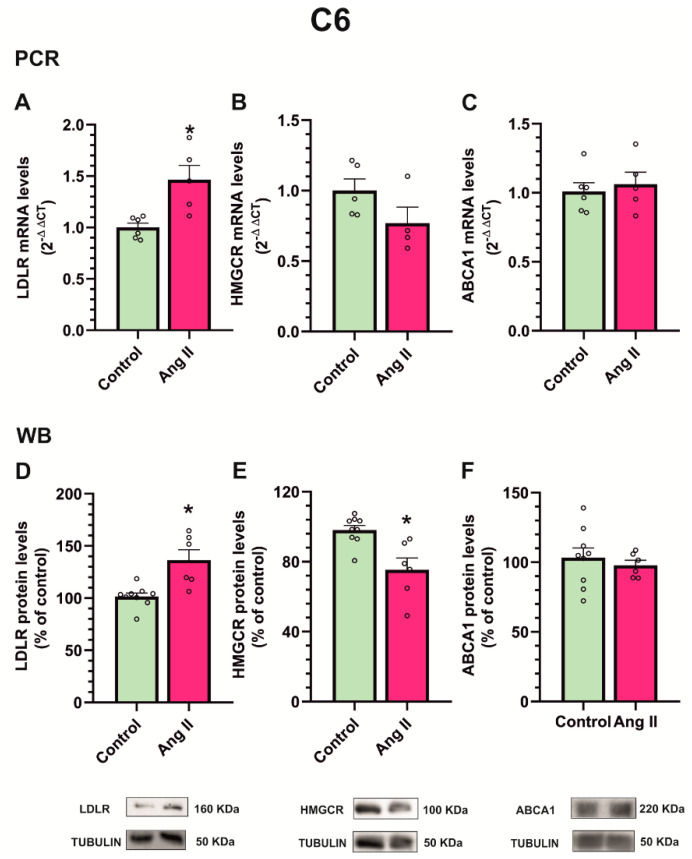
Effect of angiotensin treatment on C6 astroglial culture. Treatment of C6 astroglial cell line with angiotensin II (Ang II) for 24 h produced an increase in mRNA (**A**) and protein (**D**) cholesterol uptake-related molecule LDL receptor (LDLR) expression, a decrease in cholesterol biosynthesis enzyme HMGCR (**B**,**E**), and no changes in cholesterol efflux-related molecule ABCA1 (**C**,**F**). Data are mean ± standard error of the mean (SEM). * *p* < 0.05 compared to control (Student’s *t*-test). HMGCR, 3-hydroxy-3-methylglutaryl CoA reductase; ABCA1, ATP-binding cassette transporter-1.

Interestingly, the treatment of the C6 astroglial cell line with Ang II for 24 h produced an increase in gene expression and protein levels of the cholesterol uptake marker LDLR in the astroglial cells (Figure 10A,D) and a simultaneous decrease in the gene expression and protein levels of the cholesterol biosynthesis enzyme HMGCR (Figure 10B,E), with no significant changes in efflux marker protein ABCA1 (Figure 10C,F). Altogether, the results suggest that Ang II induces cholesterol synthesis in dopaminergic neurons and cholesterol efflux, presumably towards astrocytes, which reduce cholesterol synthesis and increase their cholesterol uptake.

In the present study, we did not perform similar in vitro experiments on mechanisms involved in Ang II/ROCK interactions because we have addressed this question in several previous studies. We have shown that Ang II/AT1 activation induces an increase in ROCK activation induced by mechanisms related to cellular oxidative stress and neuroinflammation (for details see [20,24,25]).

## 4. Discussion

In the present study, we observed that simvastatin, an inhibitor of cholesterol synthesis, induced a significant reduction in the development of LID in a rat PD model induced by the dopaminergic neurotoxin 6-OHDA, and that this was not related to any simvastatin interference with the motor improvement induced by the therapeutic effect of L-DOPA, as revealed via the analysis of the motor behavior using the cylinder and the rotation tests. Simvastatin significantly reduced all LID components from the first week of treatment until the end of the treatment (3 weeks), with the reduction in the orolingual subtype being more pronounced. Previous studies have also reported that statins can reduce dyskinesia in rat and monkey PD models without affecting the L-DOPA antiparkinsonian efficacy [28,29,30]. However, an exploratory trial in patients revealed no significant effect, suggesting that higher doses are needed [28]. These previous studies [28,29,30] suggested that the effects of statins on LID were related to a reduction in the activation of Ras-extracellular signal-regulated kinase (ERK1/2), a molecular pathway essential in the pathophysiology associated with dyskinesia [42,43], rather than with cholesterol-dependent effects. It was suggested that the development of LID is related to the repeated aberrant overactivation of ERK1/2/MAP kinase pathway in the neurons of the direct striatal pathway as the consequence of the L-DOPA activation of their D1 dopamine receptors, which show supersensitivity in the dopamine-depleted striatum [9,10,44], However, in the present study, we observed that dyskinetic rats showed a significant increase in the levels of cholesterol and the cholesterol biosynthesis enzyme HMGCR, which were inhibited by simvastatin. As observed in our previous studies, LID was also associated with an increase in levels of ROCK activity and the inflammatory marker IL-1β [15,21], which were also reduced by simvastatin. Moreover, we have previously observed that the inhibition of ROCK using fasudil inhibited the inflammatory response in the nigra and striatum and reduced LID in rats [21], and that AT1 receptor antagonists, such as candesartan, which inhibit ROCK activity [20,25], also reduced rat LID and neuroinflammation [15]. In the present study, we observed that fasudil and candesartan also inhibit the LID-related increase in the cholesterol biosynthesis enzyme HMGCR. Altogether the data suggest mutual interaction between AT1, HMGCR, and ROCK inhibition in LID reduction and L-DOPA-related inflammatory responses. Consistent with this, it has been observed that cholesterol increases inflammation by activating inflammasome [45] and increasing TNF-α and other cytokines in animal models or patients with neurodegenerative disorders [46] and that statins reduce neuroinflammation, decreasing TNF-α and IL-1β [32,33,34]. A considerable number of previous studies have shown that AT1 receptor and ROCK activation mediate the inflammatory response in the brain and peripheral tissues and that the activation of the proinflammatory prooxidative Ang II/AT1/NADPH-oxidase axis induces ROCK activation [20,26] and the activation of the NLRP3 inflammasome in the nigra and striatum [22]. Consistent with this, mutual regulation between cholesterol/statins, ROCK, and Ang II has been observed in peripheral tissues.

In peripheral tissues, statins have been observed to decrease Ang II/AT1 activity by decreasing AT1 receptor expression [47,48,49]. Conversely, Ang II induced cholesterol accumulation in podocytes, which led to podocyte damage [50]. In the present study, we observed that both Ang II and dyskinesia can induce the synthesis of cholesterol, although the results suggest that the neuronal accumulation of cholesterol may be reduced via transfer to astrocytes. It is well known that the Ang II/AT1/NADPH-oxidase pathway is a major source of ROS in cells, including neurons and glial cells [41,51]. It has been suggested that lipid, including cholesterol, accumulation in neurons may be related to ROS production as an initial neuroprotective response, although excessive accumulation may lead to neurodegeneration [52,53]. However, lipids can be initially transferred from neurons to astrocytes, which are able to accumulate and metabolize these lipids [54,55].

In addition, it is well known that Ang II induces redox-sensitive ROCK activation both in the periphery and in the brain [20,56], and we have shown mutual positive regulation between AT1/NADPH-oxidase and ROCK mediated by superoxide and p38 mitogen-activated protein kinase (p38-MAPK) in glial cells [20]. In cardiovascular tissues, statins have been shown to inhibit the increase in ROCK activity induced by Ang II [57] and that AT1 antagonists (ARBs), and statins act synergically to reduce ROCK activity and its deleterious effects on the progression of heart damage and atherosclerosis [57,58,59]. It has been suggested that statins inhibit the production of intermediates for cholesterol biosynthesis, such as farnesyl pyrophosphate and geranylgeranyl pyrophosphate, which are also essential for the post-translational modification of the Rho family proteins [60,61]. Consistent with this, different studies in vascular tissues have observed that the inhibition of Rho and ROCK through statins is a major mechanism mediating the pleiotropic effects of statins [62,63,64]. In these studies, ROCK inhibition usually required high doses of statins, and inhibition was usually lower than that observed by the direct use of ROCK inhibitors such as fasudil [63]. Although all drugs may have side effects in some patients, in the case of statins, the possibility of statin-associated muscle symptoms (SAMS) should be particularly considered in PD patients characterized by major movement disorders. In the present short-term experiments, simvastatin induced a significant reduction in the development of LID without any interference with the motor improvement induced by the therapeutic effect of L-DOPA. However, the potential neuromuscular adverse effects of statins in PD patients under chronic treatment may be expected, as observed in patients treated with statins for different diseases [65,66].

Angiotensin receptors, ROCK, and cholesterol have been identified in different peripheral cell types and throughout the brain, including substantia nigra, striatum, and in neurons and glial cells, as we confirmed using the laser microdissection of isolated cells and other methods [25,67,68]. However, interactions between RAS, ROCK, and dopamine are particularly interesting, as the RAS and the dopaminergic system counter regulate each other, both at the level of the nigrostriatal system and in peripheral renal and cardiovascular systems. The dysregulation of RAS/dopamine interactions leads to the exacerbation of inflammation and cell degenerative processes, both in the brain and peripheral organs (see the review by Labandeira-Garcia et al. [18,26,51]). Fluctuations in dopamine levels in the nigrostriatal system characterize LID, together with dysregulations in the above-mentioned pro-inflammatory parameters, which are attenuated by the corresponding inhibitor. Furthermore, in the present study, we observed interactions between simvastatin, AT1 antagonists, and ROCK inhibitors in the substantia nigra and striatum of rats with LID, which may explain why the three treatments reduce dyskinesia. The results show that these interactions were similar in the two major regions related to LID (i.e., substantia nigra and striatum) that were studied simultaneously in the present experiments. The dysregulations of Angiotensin/AT1 activity, ROCK activity, and cholesterol homeostasis interact with each other and are involved in neuroinflammatory responses related to LID. Consistent with this, we observed that markers of neuroinflammation, such as IL-1β, were increased by LID both in the nigra and striatum, which was inhibited by simvastatin. In previous studies, we also showed that the expression of other neuroinflammatory markers, such as TNF-α, was inhibited by AT1 blockers and ROCK inhibitors [20,24]. However, ROCK inhibition appears as a final common pathway, and the direct use of ROCK inhibitors, such as fasudil, appears to be the most potent strategy. It was reasonable to think that the combination of simvastatin with fasudil may facilitate a reduction in effective doses of simvastatin necessary to induce a significant reduction in LID in humans. However, the present experiments in rats treated with simvastatin and low doses of fasudil did not reveal a significant improvement relative to simvastatin alone or low doses of fasudil alone.

Interestingly, the three types of drugs (ARBs, statins, and ROCK inhibitors) have also been suggested as neuroprotective treatments against dopaminergic degeneration and PD, through mechanisms mainly related to the above-mentioned reduction in oxidative stress and neuroinflammation. The neuroprotective properties of ARBs have been shown in PD models [19,51,69] and clinical studies [70,71]. The neuroprotective effects of ROCK inhibitors have been shown in different PD models [25,72]. The neuroprotective effects of statins have been reported in different studies [34,73], and reductions in neuroinflammation [33], oxidative stress [74] and alpha-synuclein aggregation [75] have been suggested as possible neuroprotective mechanisms for the three types of drugs.

## 5. Conclusions

The results suggest mutual interaction between angiotensin/AT1, cholesterol, and ROCK pathways in LID, which are reduced by the corresponding inhibitors. Interestingly, the three types of drugs have also been suggested as neuroprotective treatments against Parkinson’s disease. Therefore, they may reduce dyskinesia and the progression of disease sharing common mechanisms.

## Data Availability

The datasets used and/or analyzed during the current study are available from the corresponding author on reasonable request.
